# Factors Influencing the Conversion of Ocular Myasthenia Gravis to Generalized Myasthenia Gravis: A Retrospective Cohort Study

**DOI:** 10.1155/joph/6652248

**Published:** 2026-02-11

**Authors:** Wanicha Chuenkongkaew, Niphon Chirapapaisan, Pawimon Chatchutimakorn, Natthapon Rattanathamsakul, Manassawee Joradoln, Pawita Kongthanasomboon, Akarawit Eiamsamarng

**Affiliations:** ^1^ Department of Ophthalmology, Faculty of Medicine Siriraj Hospital, Mahidol University, Bangkok, Thailand, mahidol.ac.th; ^2^ Department of Medicine, Faculty of Medicine Siriraj Hospital, Mahidol University, Bangkok, Thailand, mahidol.ac.th; ^3^ Department of Ophthalmology, Faculty of Medicine Siriraj Hospital, Golden Jubilee Medical Center, Mahidol University, Bangkok, Thailand, mahidol.ac.th

**Keywords:** generalized myasthenia gravis, myasthenia gravis, myasthenia gravis progression, ocular myasthenia gravis, thymic abnormalities

## Abstract

**Purpose:**

The conversion rate of ocular myasthenia gravis (OMG) to generalized myasthenia gravis (GMG) lacks definitive predictors.

**Methods:**

This retrospective cohort study analyzed data collected at Siriraj Hospital between January 2007 and December 2019 to identify factors influencing OMG generalization and the time to conversion. The records of 200 OMG patients were reviewed and both acetylcholine receptor antibody (AChR Ab)‐positive and AChR Ab‐negative patients were included.

**Results:**

Seventy‐eight (39%) developed GMG, with a median conversion time of 16 months (IQR 7.88, 33.75) and a 2‐year conversion rate of 25.5%. AChR Ab positivity (adjusted HR 2.88, 95% CI 1.79–4.63), thymic abnormalities (adjusted HR 2.30, 95% CI 1.41–3.74), smoking (adjusted HR 1.78, 95% CI (1.04, 3.03), and pyridostigmine dosages > 180 mg/day (adjusted HR 2.33, 95% CI 1.41–3.87) were significantly associated with shorter conversion time.

**Conclusion:**

Thymic abnormalities and positive AChR Ab warrant routine assessment in all OMG patients. Smoking cessation is crucial, as it may impact conversion risk and time. Unlike previous findings suggesting a protective role of pyridostigmine, our data indicate a strong association between high‐dose pyridostigmine and conversion to GMG, likely reflecting underlying disease severity. This underscores the need for individualized risk assessment in OMG management.

## 1. Introduction

Ocular myasthenia gravis (OMG) is an autoimmune disease mediated by acetylcholine receptor antibodies (AChR Ab) that target the postsynaptic neuromuscular junction. This process selectively affects the extraocular muscles, orbicularis oculi, and levator palpebrae superioris, causing fatigable ptosis and/or binocular diplopia [1–3]. Fluctuation and fatigable weakness are hallmarks of this condition.

There are noticeable geographical differences in the clinical manifestations, incidence, and prevalence of OMG, as well as in the age at onset [4]. Within 2 years of ocular symptom onset, up to 80% of OMG patients progress to generalized myasthenia gravis (GMG), which involves bulbar, limb, and respiratory muscle weakness in addition to ptosis and diplopia. There is extensive global variability in the conversion rate to GMG [1, 5].

Several factors, including age at symptom onset, thymic abnormalities, positive AChR Ab results, cigarette smoking, and positive repetitive nerve stimulation (RNS) test results, have been associated with OMG conversion to GMG [1, 6]. Studies in Singapore and South Korea indicate that positive RNS tests and anti‐AChR Ab positivity are associated with the conversion of OMG to GMG [4, 7]. Furthermore, a German study suggested that thymoma is a predictive factor for conversion [8]. Various studies have shown that treatment with immunosuppressive agents (corticosteroids) reduces the conversion rate to GMG [1, 4, 7, 9, 10].

Although several factors have been reported, definitive predictive factors for the generalization of OMG remain incompletely understood, underscoring the need for validation in larger cohorts and the investigation of additional factors. Most prior studies, particularly in Asia, have been limited by small sample sizes, relatively narrow inclusion criteria (e.g., only AChR Ab‐positive patients), or short follow‐up periods. In addition, the effect of treatment dose, such as pyridostigmine dosage, has not been adequately explored.

To address these gaps, we conducted a retrospective cohort study using a larger and more diverse OMG population. Our cohort includes both seropositive and seronegative patients and spans a 13‐year follow‐up period. This study aimed to evaluate the risk and protective factors associated with the conversion of OMG to GMG and to identify the factors influencing the time to conversion. Our findings may provide valuable insights to help prevent OMG progression to GMG.

## 2. Methods

This retrospective cohort study analyzed the data of patients diagnosed with OMG at Siriraj Hospital, a tertiary care center in Bangkok, Thailand. The data were collected between January 2007 and December 2019, and the data were reviewed by one investigator in the neuro‐ophthalmology division. Institutional ethics approval was obtained from the Siriraj Institutional Review Board, and the study fully complied with the Declaration of Helsinki (COA No. Si631‐2021). Patient informed consent was waived by the ethics committee.

The eligibility criteria for the study required patients to have an OMG diagnosis with initial presentation limited to ocular symptoms (ptosis or diplopia) and to meet all of the following criteria.

• Fluctuating and fatigable weakness of extrinsic ocular muscles (ptosis or binocular diplopia).
• At least one positive diagnostic test result: AChR Ab, neostigmine test, abnormal RNS (amplitude decrement > 10%), increased jitter in a single‐fiber electromyography test, or a positive ice/fatigue test demonstrating a clinical response to pyridostigmine.
• Diagnosis of OMG by a neuro‐ophthalmologist or neurologist.

The ice test was deemed positive if either ≥ 2‐mm ptosis improvement occurred after a 2**-**min ice pack application or if a ≥ 50% reduction in ocular deviation (by alternate prism cover test) was observed after a 5‐min ice pack application. A positive fatigue test indicated fatigable ptosis after sustained upgaze for 1 min.

The exclusion criteria were congenital or juvenile OMG, initial presentation with GMG symptoms, incomplete records regarding OMG or GMG onset, incomplete medical data, prior eyelid or strabismus surgery, and thyroid‐associated ophthalmopathy.

All OMG patients were monitored until either GMG conversion occurred or their last follow‐up visit. GMG conversion was diagnosed when patients developed limb, axial, or facial muscle weakness (distinct from ocular muscles); bulbar symptoms (difficulty swallowing, chewing, speaking, hoarseness); or respiratory difficulty/failure. Experienced neurologists made all the diagnoses.

Clinical and demographic data were extracted from Siriraj Hospital’s electronic medical records. The data included initial treatment date, sex, underlying disease, smoking history, patient age at OMG onset, initial ocular symptoms (ptosis, diplopia), OMG treatment before conversion (pyridostigmine, prednisolone), thymic abnormalities, and GMG symptom onset. Chest computed tomography or pathological findings were used to reveal thymic abnormalities. Pyridostigmine dosage was recorded as the maximum dosage that continued to be used under disease control. Additionally, we collected the maximum dosage of immunosuppressive treatments (prednisolone, azathioprine, MMF, and CSA) and the time to start immunosuppression after diagnosis. After enrollment, patients were categorized into either a “conversion group” (patients who had converted from OMG to GMG) or a “nonconversion group” (the remaining OMG patients).

### 2.1. Statistical Analysis

PASW Statistics Version 18 (SPSS Inc, Chicago, IL, USA) was used for statistical analysis. The demographic and clinical characteristics were summarized using descriptive statistics (means, standard deviations, and percentages). Medians and interquartile ranges (IQRs) reported the following durations: OMG onset to GMG, OMG onset to first treatment, and follow‐up (from OMG onset to last visit or GMG symptoms). Categorical variables were analyzed with the chi‐square test, and continuous variables were analyzed with the unpaired *t*‐test.

Cox proportional regression analysis was used to evaluate the association between risk factors and the time to GMG conversion. Multivariate analysis was performed for variables with a *p* value < 0.2 from a log‐rank test. Logistic regression and Cox proportional hazard models assessed risk factors, including sex, smoking history, thymic abnormalities, AChR Ab, prednisolone (an immunosuppressive agent), and pyridostigmine. Kaplan–Meier survival curves explored the relationship between the time from OMG onset to GMG and demographic, clinical, manifestation, investigation, and treatment variables.

## 3. Results

**FIGURE 1:**
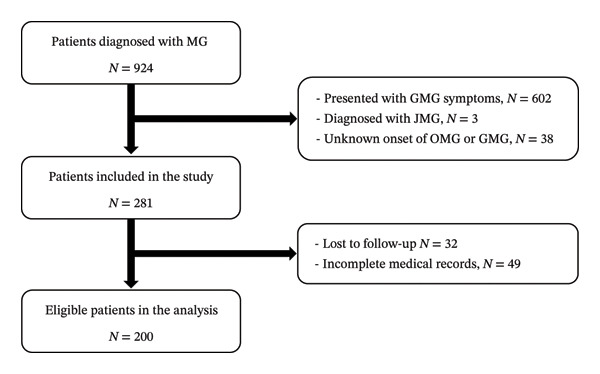
Participant flowchart for the ocular myasthenia gravis study. *GMG* generalized myasthenia gravis, *JMG* juvenile myasthenia gravis, *MG* myasthenia gravis, *OMG* ocular myasthenia gravis.

An initial review of the patients’ electronic medical records revealed 924 patients diagnosed with myasthenia gravis between January 2007 and December 2019. We excluded 602 patients who initially presented with GMG symptoms, 3 with juvenile myasthenia gravis, 38 with incomplete records on OMG/GMG onset, 32 lost to follow‐up, and 49 with incomplete medical data (Figure 1). This study analyzed 200 patients with a confirmed OMG diagnosis at Siriraj Hospital (January 2007–December 2019) who met the inclusion criteria and had complete medical records. Of these, 78 (39%) progressed to GMG, while 122 (61%) remained in the nonconversion group at the conclusion of the study. The median follow‐up time was 63.5 months (IQR 31, 107.5) for the nonconversion group and 16 months (IQR 7.88, 33.75) for the conversion group. Table 1 summarizes the participant demographics and clinical characteristics. Over 60% of participants were female, which was consistent across the OMG (61.5%) and GMG (67.9%) groups. The mean age at onset of OMG was 49.17 years (SD 15), with 48.35 years (SD 15.26) in the OMG group and 50.44 years (SD 14.55) in the GMG group.

**TABLE 1:** Clinical characteristics of participants in the ocular myasthenia gravis study.

Characteristics	Total (*N* = 200)	Nonconversion group (*n* = 122)	Conversion group (*n* = 78)	*p* value
Age at onset of OMG, mean (SD)	49.17 (14.99)	48.35 (15.26)	50.44 (14.55)	0.339
Sex, *n* (%)				0.352
Male	72 (36)	47 (38.5)	25 (32.1)	
Female	128 (64)	75 (61.5)	53 (67.9)	
History of smoking, *n* (%)	36 (18)	17 (13.9)	19 (24.4)	0.061
Ocular symptoms, *n* (%)				
Ptosis	182 (91)	108 (88.5)	74 (94.9)	0.126
Diplopia	113 (56.5)	66 (54.1)	47 (60.3)	0.392
Duration of symptom onset to the first visit (mo), median (IQR)	2 (11.5)	3 (11)	1 (5.5)	**0.007**
Presence of thymic abnormalities, *n* (%)	45 (22.5)	13 (10.7)	32 (41)	**< 0.001**
Positive anti‐AChR Ab, *n* (%)	61 (30.5)	20 (16.4)	41 (52.6)	**< 0.001**
Positive test of EMG, *n* (%)	31 (63.27) (*n* = 49)	12 (42.86) (*n* = 28)	19 (90.48) (*n* = 21)	**< 0.001**
Immunosuppressive treatment				
Prednisolone				
*n* (%)	133 (66.5)	63 (51.64)	70 (89.74)	**< 0.001**
Max dose (mg/d), mean (SD)	30.81 (12.42)	29.76 (12.93)	31.75 (11.96)	0.179
Time to start from OMG diagnosed (mo), median (IQR)	2.43 (14.19) (*n* = 52)	1.85 (5.24) (*n* = 24)	3.83 (17.09) (*n* = 28)	0.186
Azathioprine				
*n* (%)	64 (32)	18 (14.75)	46 (58.97)	**< 0.001**
Max dose (mg/d), mean (SD)	76.95 (31.59)	63.89 (28.73)	82.07 (31.47)	**0.019**
Time to start from OMG diagnosed (mo), median (IQR)	32.10 (34.45) (*n* = 60)	23.75 (38.33) (*n* = 16)	32.18 (48.1) (*n* = 44)	0.514
Pyridostigmine dosage, *n* (%)				**< 0.001**
≤ 180 mg/day	170 (85)	114 (93.4)	56 (71.8)	
> 180 mg/day	30 (15)	8 (6.6)	22 (28.2)	

*Note:* anti‐AChR, Ab, anti–acetylcholine receptor antibodies; IQR, interquartile range; EMG, electromyography (including RNS and/or SF‐EMG); mo, months. Statistically significant *p* values are shown in bold (*p* < 0.05).

Abbreviations: OMG, ocular myasthenia gravis; SD, standard deviation.

Immunosuppressive therapy was initiated in all patients who developed GMG, as well as in OMG patients who exhibited disabling diplopia or ptosis unresponsive to pyridostigmine. The treatment was also started in OMG patients considered at high risk for GMG, including those with positive AChR antibodies, abnormal thymic imaging, or those requiring high doses of pyridostigmine for symptom control. Prednisolone was the first‐line immunosuppressive agent in 133 patients. Additional immunosuppressants, such as azathioprine and mycophenolate mofetil (MMF), were used in 71 patients due to clinical worsening despite steroid therapy (*n* = 43), inability to taper steroids (*n* = 12), steroid‐related side effects (*n* = 5), and others. Azathioprine was the most commonly used adjunctive agent (*n* = 66), while MMF was used in five patients due to initiation at another institution (*n* = 4) or high genetic risk for azathioprine intolerance (*n* = 1). Two patients on azathioprine were switched to MMF due to azathioprine‐induced hepatitis, and one patient was switched to cyclosporine because of comorbid focal segmental glomerulosclerosis.

Several factors correlated with the time to conversion (Table 2). These were a history of smoking, thymic abnormalities, positive AChR Ab, and pyridostigmine dosages greater than 180 mg/day. The adjusted hazard ratios, calculated using a multivariate Cox proportional hazard model, showed significant associations with the progression from OMG to GMG. Specifically, for a history of smoking, thymic abnormalities, positive AChR Ab, and pyridostigmine dosages exceeding 180 mg/day, the adjusted hazard ratios were 1.78 (95% CI 1.04–3.03), 2.30 (95% CI 1.41–3.74), 2.88 (95% CI 1.79–4.63), and 2.33 (95% CI 1.41–3.87), respectively. Conversely, our analysis revealed that treatment with prednisolone before conversion did not significantly reduce the risk of progression to GMG.

**TABLE 2:** Hazard ratios for conversion from ocular myasthenia gravis to generalized myasthenia gravis.

Variable	Crude HR (95% CI)	*p* value	Adjusted HR (95% CI)	*p* value
Sex^a^	1.21 (0.75, 1.95)	0.430	—	—
History of smoking	1.83 (1.09, 3.07)	0.023	1.78 (1.04, 3.03)	**0.035**
Thymic abnormalities	3.65 (2.30, 5.79)	< 0.001	2.30 (1.41, 3.74)	**0.001**
Positive anti‐AChR Ab	3.48 (2.22, 5.45)	< 0.001	2.88 (1.79, 4.63)	**< 0.001**
Pyridostigmine dosage^b^	2.77 (1.69, 4.54)	< 0.001	2.33 (1.41, 3.87)	**0.001**
Usage of prednisolone before conversion	0.71 (0.45, 1.13)	0.152	0.67 (0.42, 1.08)	0.098
Duration from onset of OMG to first pyridostigmine treatment	0.60 (0.38, 0.94)	0.026	0.86 (0.54, 1.37)	0.528

*Note:* AChR Ab, acetylcholine antibody. Statistically significant *p* values are shown in bold (*p* < 0.05).

Abbreviations: CI, confidence interval; HR, hazard ratio; OMG, ocular myasthenia gravis.

^a^Sex with reference of male.

^b^Pyridostigmine dosage with reference of ≤ 180 mg/day.

However, the subgroup analysis of AChR Ab‐positive patients (*n* = 61) showed no significant association between sex (HR 0.96, 95% CI 0.51–1.80, *p* = 0.898), history of smoking (HpR 1.71, 95% CI 0.83–3.56, *p* = 0.166), thymic abnormalities (HR 1.57, 95% CI 0.84–2.96, *p* = 0.167), or pyridostigmine dosage exceeding 180 mg/day (HR 1.26, 95% CI 0.55–2.89, *p* = 0.594) with progression to GMG in univariate analysis (Supporting table) (available here).

The relationship between pyridostigmine dosage and ptosis severity was assessed by categorizing patients based on pretreatment MRD1: mild (2–3.5 mm), moderate (0.5–1.5 mm), and severe (≤ 0 mm) ptosis. Pyridostigmine doses were grouped into ≤ 180 mg/day and > 180 mg/day. Fisher’s exact test revealed no significant association between pyridostigmine dose and ptosis severity (*p* = 0.420). The distribution of doses was similar across the severity groups: 11.1% of mild, 5.6% of moderate, and 16.9% of severe ptosis patients received doses > 180 mg.

Of the 200 patients, 78 (39%) progressed to GMG during follow‐up, with a median conversion time of 16 months (IQR 7.88, 33.75). Factors associated with a shorter median conversion time were the following.

• Smoking status (42 months).
• Thymic abnormalities (26 months).
• Positive AChR Ab (33 months).
• Pyridostigmine dosages > 180 mg/day (30 months).
• A time from OMG onset to initial pyridostigmine ≤ 1 month (120 months).

**FIGURE 2:**
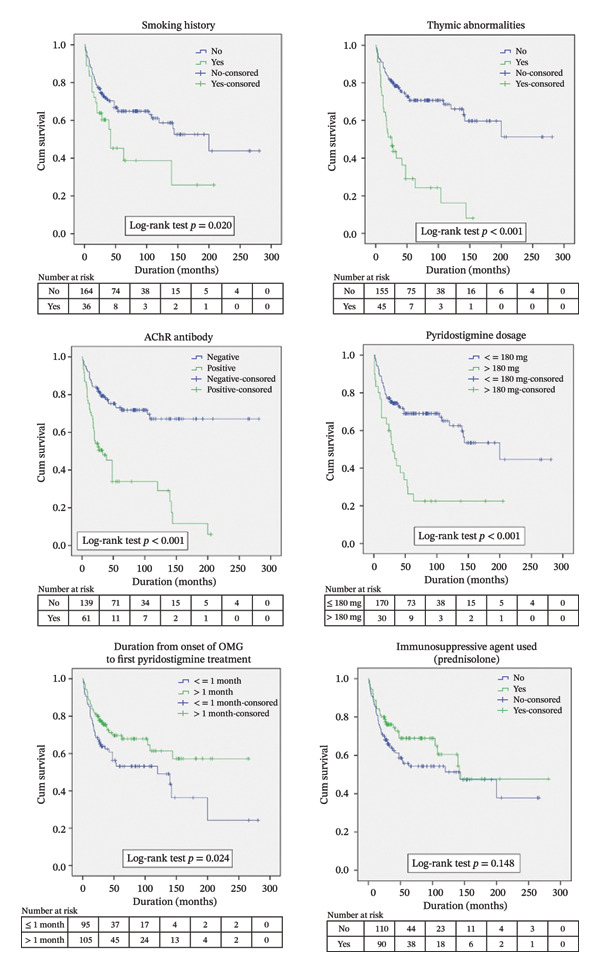
Kaplan–Meier analysis of time‐to‐event data for factors in ocular myasthenia gravis conversion to generalized myasthenia gravis.

Immunosuppressive therapy (prednisolone) was not significantly associated with a longer median conversion time (142 months). Figure 2 (Kaplan–Meier curve) shows the time‐to‐event data for each factor.

The likelihood of progression from OMG to GMG at 2, 4, and 6 years was 26%, 34%, and 39%, respectively. The analysis identified several factors associated with an increased likelihood of conversion. These factors included smoking, thymic abnormalities, positive AChR Ab, pyridostigmine dosages exceeding 180 mg/day, and the initiation of pyridostigmine treatment within 1 month of OMG onset. However, the receipt of immunosuppressive agents, as assessed by the log‐rank test (*p* = 0.148), did not significantly decrease the probability of conversion at 2, 4, or 6 years (Table 3).

**TABLE 3:** Median conversion time and probability of conversion to generalized myasthenia gravis associated with risk factors.

Risk factor	Median conversion time, mo (SE)	Probability of conversion (SE)		
		2 y	4 y	6 y
Overall	144 (31.33)	0.26 (0.03)	0.34 (0.04)	0.39 (0.04)
Sex				
Male	142 (NA)	0.22 (0.05)	0.32 (0.06)	0.35 (0.06)
Female	144 (37.13)	0.27 (0.04)	0.35 (0.04)	0.42 (0.05)
Smoking				
Yes	42 (14.21)	0.36 (0.08)	0.55 (0.10)	0.61 (0.10)
No	200 (46.30)	0.23 (0.03)	0.30 (0.04)	0.35 (0.04)
Thymic abnormalities				
Yes	26 (6.22)	0.49 (0.075)	0.64 (0.08)	0.76 (0.08)
No	NA	0.19 (0.031)	0.25 (0.04)	0.29 (0.04)
Anti‐AChR Ab				
Positive	33 (8.45)	0.46 (0.06)	0.55 (0.07)	0.66 (0.07)
Negative	NA	0.17 (0.03)	0.25 (0.04)	0.28 (0.04)
Pyridostigmine dosage				
≤ 180 mg/day	200 (46.97)	0.23 (0.03)	0.28 (0.04)	0.31 (0.04)
> 180 mg/day	30 (4.98)	0.40 (0.09)	0.66 (0.09)	0.78 (0.08)
Treatment with prednisolone				
Yes	142 (NA)	0.20 (0.04)	0.27 (0.05)	0.31 (0.05)
No	144 (52.79)	0.30 (0.04)	0.39 (0.05)	0.46 (0.05)
Duration from onset of OMG to first pyridostigmine treatment				
≤ 1 mo	120 (46.50)	0.32 (0.05)	0.39 (0.05)	0.47 (0.06)
> 1 mo	NA	0.20 (0.04)	0.30 (0.05)	0.32 (0.05)

Abbreviations: OMG, ocular myasthenia gravis; SE, standard error.

## 4. Discussion

This study aimed to identify the risk and protective factors associated with patients’ progression from OMG to GMG. The demographic data revealed a mean age at onset for OMG of 49.17 years, aligning with findings from prior research in Asia [6, 7], although some Asian studies have documented an older age at onset [1, 4]. Our research also revealed a female predominance, consistent with the findings of studies in Asia and Germany [1, 6, 8, 11] but contrasting with research in Argentina [12].

This analysis indicated a conversion rate of 25.5% at 2 years after onset, which parallels the outcomes of studies within Asian populations [1, 4, 6, 7]. However, the conversion rates reported in some Western studies are nearly double those found in Asia [8, 12–14]. Several factors likely contribute to the variability in conversion rates. These include differences in demographic data, the absence of uniform diagnostic criteria, and the use of diverse diagnostic tests. Additionally, variations in sex distribution, age at OMG onset, and the widespread use of immunosuppressive agents to prevent OMG progression to GMG may also play a role [6, 15]. Moreover, an elevated risk of conversion to GMG has been documented in individuals of younger age and female sex, characteristics that were predominant among our patient cohort [1, 6, 14].

The findings of our study highlighted thymic abnormalities and positive AChR Ab results as substantial risk factors for the conversion from OMG to GMG, corroborating the results of previous research [1, 4, 7, 8, 12–14, 16–18]. This association is predicated on the hypothesis that the pathophysiology of myasthenia gravis involves an abnormal thymus that produces AChR Ab. Therefore, chest imaging and testing for anti‐AXhR Ab should be conducted for all patients diagnosed with OMG. Such measures aim to facilitate early prevention strategies and reduce the conversion rate to GMG in patients demonstrating thymic abnormalities or positive anti‐AChR Ab results, aligning with insights into the disease process.

A recent study posited that pyridostigmine administration could lower the conversion rate to GMG due to its immunomodulatory effects on the cholinergic anti‐inflammatory pathway [1]. In contrast, our analysis found that pyridostigmine dosages > 180 mg/day were significantly associated with a shorter time to conversion. The findings could be attributed to the necessity of administering higher dosages of pyridostigmine in patients who present with more severe OMG symptoms. These patients may experience a diminished response to pyridostigmine treatment, potentially increasing their risk of conversion to GMG [2]. Although Fisher’s exact test showed no significant association between pyridostigmine dosage and ptosis severity, the possibility of confounding by disease severity remains important, as higher doses are typically prescribed to patients with more severe OMG symptoms who may have a diminished response to treatment. Therefore, while our data show a strong association, we caution that pyridostigmine dosage may serve as a marker of more severe disease rather than an independent risk factor for generalization.

Our Cox proportional hazard model revealed a significant correlation between smoking and a shorter time to GMG conversion in overall cohort and subgroup analysis for AChR‐Ab‐positive patients showed an even stronger association. These findings are consistent with previous research [1], which suggested that smoking heightened the risk of transitioning from OMG to GMG in patients seropositive for AChR Ab. This association could be elucidated by two hypotheses.

First, smoking exerts many effects on the immune system, including the recruitment of inflammatory cells and the subsequent release of inflammatory cytokines and matrix metalloproteinases, which are enzymes that contribute to tissue damage [19]. These mechanisms, among others, underlie the association between smoking and various autoimmune diseases, such as rheumatoid arthritis, systemic lupus erythematosus, multiple sclerosis, and Graves’ disease [19, 20]. Given the established link between smoking and an increased risk of more severe ophthalmopathy in Graves’ disease [21], it is plausible that smoking may influence the pathogenesis of OMG. Due to the coexistence of Graves’ disease and OMG, smoking might exacerbate ocular symptoms in OMG patients [1, 22].

Second, the postsynaptic acetylcholine receptors targeted by the anti‐AChR Ab in myasthenia gravis patients become desensitized due to prolonged exposure to blood nicotine [23]. This can lead to a blockade at the neuromuscular junction, exacerbating symptoms in OMG patients and increasing the likelihood of conversion to GMG [1, 23].

Based on these insights, we recommend that patients with myasthenia gravis cease smoking to mitigate the risk of progression to GMG and to ameliorate the severity of symptoms associated with OMG and GMG.

Multiple studies have documented that immunosuppressive agents, including oral corticosteroids, can reduce the conversion rate from OMG to GMG and delay the onset of generalization [1, 6–8, 16, 24]. However, the ability of prednisolone to delay the conversion from OMG to GMG was not significantly different according to our multivariate Cox proportional hazard model.

In contrast to a previous large cohort study which suggested that male sex was associated with a reduced risk of progression from OMG to GMG [25], our study did not find sex to be significantly associated with conversion. Additionally, a recent systematic review that pooled data from 31 studies found only a slight, statistically nonsignificant increased risk for females [26]. These findings suggest that there is insufficient evidence to conclude that sex is a risk factor for conversion. The observed discrepancy may be attributed to differences in study design, sample size, or cohort characteristics.

The subgroup analysis of AChR Ab‐positive patients (*n* = 61) did not show significant association between the examined factors—smoking history, thymic abnormalities, and pyridostigmine dosage > 180 mg/d—and progression from OMG to GMG. This may be due to the small sample size, which limits statistical power. It is also possible that the presence of AChR antibodies plays a dominant role in disease progression, overshadowing the impact of other factors. Given the sample size limitations, larger studies are needed to better understand the factors influencing progression in this group.

By analyzing the data from 924 patients before exclusion, we uncovered an unexpected finding: 602 patients (74.89%) initially presented with GMG, which is in marked contrast to the commonly cited figures of 20% for GMG and 80% for OMG. We attribute this discrepancy to our hospital being a tertiary care center. Patients with severe GMG symptoms are referred to the hospital, resulting in a higher prevalence of GMG than typically reported.

The strengths of this study lie in its substantial cohort size within an Asian population (*N* = 200). By including OMG patients with positive and negative AChR Ab results, our study’s findings have broad applicability to the local patient population. It is important to note that patients with negative AChR Ab test results received a confirmed diagnosis of OMG through alternative diagnostic methods. These methods include RNS, clinical tests (e.g., ice test and sleep test), and response to pyridostigmine, all of which are associated with higher rates of false positives. Furthermore, we introduced a novel analysis of pyridostigmine dosage and its potential association with generalization, which has not been evaluated in prior work.

The limitations of this study include its retrospective design, which resulted in missing or incomplete old medical records and a relatively short follow‐up period, although we consider 6 months to be sufficient for evaluation. Additionally, the historical unavailability of the test for anti‐MuSK antibodies in Thailand limited the number of patients tested, leading to a lack of data for analyzing the relationship between anti‐MuSK antibodies and conversion rates from OMG to GMG. Furthermore, we could not assess certain factors, such as autoimmune and thyroid diseases, due to the small number of patients presenting with these conditions. Moreover, only a few OMG patients underwent RNS and single‐fiber electromyography tests. Another important limitation of this study is the potential selection bias, as the cohort was derived from a tertiary referral center, with 65% of patients initially presenting with GMG excluded. This may result in a cohort that represents a specific subset of OMG patients, potentially with a different risk profile than the broader OMG population encountered in community settings. As such, the generalizability of the findings, including the observed conversion rate and risk factors, to nontertiary populations should be interpreted with caution.

In summary, our findings expand on previous research by incorporating a larger and more diverse cohort, analyzing novel predictors. This study observed a 25.5% conversion rate from OMG to GMG within 2 years after onset. Thymic abnormalities and a positive AChR Ab test result were found to be significant risk factors strongly associated with progression to GMG. Hence, evaluating for thymic abnormalities and conducting AChR Ab tests should be standard practices for all OMG patients. Smoking was identified as an additional risk factor that not only increases the probability of conversion to GMG but also accelerates the conversion timeline. Emphasizing the importance of smoking cessation could substantially ameliorate OMG symptoms and mitigate the risk of conversion to GMG. The use of an immunosuppressive agent, specifically oral prednisolone, appeared to offer a protective effect against generalization in patients with OMG. Therefore, patients should be comprehensively counseled on the risks and benefits of immunosuppressive therapy as an effective strategy to lower the conversion rate to GMG. Future research efforts should be directed toward executing randomized controlled trials or prospective studies to substantiate our findings.

**Nomenclature**
AChR Ab: Acetylcholine receptor antibodies
GMG: Generalized myasthenia gravis
IQR: Interquartile range
OMG: Ocular myasthenia gravis
RNS: Repetitive nerve stimulation

## Author Contributions

Wanicha Chuenkongkaew: conceptualization, data curation, formal analysis, investigation, methodology, project administration, resources, supervision, validation, visualization, writing–original draft, and writing–review and editing; Niphon Chirapapaisan: resources, supervision, and writing–review and editing; Pawimon Chatchutimakorn: resources, supervision, and writing–review and editing; Natthapon Rattanathamsakul: supervision and writing–review and editing; Manassawee Joradoln: supervision and writing–review and editing; Pawita Kongthanasomboon: writing–review and editing and project administration; Akarawit Eiamsamarng: conceptualization, data curation, formal analysis, validation, visualization, writing–original draft, writing–review and editing, and project administration.

## Funding

This research project was supported by Siriraj Research Development Fund (Grant Number (IO) R016531030), Faculty of Medicine Siriraj Hospital, Mahidol University.

## Disclosure

This study was presented as an oral presentation at the 2024 Annual Scientific Meeting/Hong Kong Ophthalmological Symposium held in conjunction with the Asian Neuro‐Ophthalmology Society (ASNOS) and the Asia Pacific Society of Ocular Oncology and Pathology (APSOOP).

## Conflicts of Interest

The authors declare no conflicts of interest.

## Supporting Information

Supporting table: Hazard ratios for conversion from ocular myasthenia gravis to generalized myasthenia gravis in AChR Ab‐positive patients.

## Supporting information


**Supporting Information** Additional supporting information can be found online in the Supporting Information section.

## Data Availability

The datasets generated and analyzed during the current study are available from the corresponding author upon reasonable request.
