# Supplementary material for: Factors Influencing the Conversion of Ocular Myasthenia Gravis to Generalized Myasthenia Gravis: A Retrospective Cohort Study
Source: J Ophthalmol. 2026 Feb 11;2026:6652248. doi: 10.1155/joph/6652248 (PMC12892196; doi:10.1155/joph/6652248)
Supplement: Supplementary file 1 — Supporting Information Additional supporting information can be found online in the Supporting Information section. [file JOPH-2026-6652248-s001.docx]

**Supplement table** Hazard ratios for conversion from ocular myasthenia gravis to generalized myasthenia gravis in AChR Ab-Positive Patients.

| **Variable** | **Crude HR (95% CI)** | ***P-value*** |
| --- | --- | --- |
| Sex^a^ | 0.96 (0.51, 1.80) | 0.898 |
| History of smoking | 1.71 (0.83, 3.56) | 0.166 |
| Thymic abnormalities | 1.57 (0.84, 2.96) | 0.167 |
| Pyridostigmine dosage^b^ | 1.26 (0.55, 2.89) | 0.594 |
| Usage of prednisolone before conversion | 0.86 (0.47, 1.60) | 0.638 |

*AchR Ab* acetylcholine antibody, *CI* confidence interval, *HR* hazard ratio. Statistically significant P-values are shown in bold (P<0.05).

^a^ Sex with reference of male

^b^ Pyridostigmine dosage with reference of ≤180 mg/day
